# *catena*-Poly[[(triphenyl­phosphane-κ*P*)copper(I)]-di-μ-bromido-[(triphenyl­phos­phane-κ*P*)copper(I)]-μ-1,3-bis(pyridin-4-yl)­propane-κ^2^ *N*:*N*′]

**DOI:** 10.1107/S1600536812004084

**Published:** 2012-02-04

**Authors:** Wenjiang Huang, Jinfang Zhang, Chi Zhang

**Affiliations:** a Institute of Molecular Engineering and Advanced Materials, School of Chemical Engineering, Nanjing University of Science and Technology, 200 Xiaolingwei, Nanjing 210094, Jiangsu, People’s Republic of China; b Institute of Science and Technology, Jiangsu University, 301 Xuefu Road, Zhenjiang 212013, People’s Republic of China

## Abstract

Through a diffusion reaction, cuprous bromide, triphenyl­phosphane and 1,3-bis­(pyridin-4-yl)propane (bpp) were self-assembled to form the one-dimensional title compound, [Cu_2_Br_2_(C_13_H_14_N_2_)(C_18_H_15_P)_2_]_*n*_. Each Cu^I^ atom is coordinated by two Br atoms, one P atom from a triphenyl­phosphane ligand and one N atom from a bpp mol­ecule in a distorted tetra­hedral geometry. Two μ_2_-Br bridges connect two [Cu(PPh_3_)]^+^ units to form neutral [CuBr(PPh_3_)]_2_ dimers, which are linked by the flexible bridging bpp ligands to form a one-dimensional chain structure parallel to the *c* axis. The dihedral angle between the pyridine rings of the bpp ligand is 34.59 (14)°.

## Related literature

For background to architectures, topologies and applications of metal–organic compounds, see: Eddaoudi *et al.* (2001 ▶); Banerjee *et al.* (2008 ▶); Zhang *et al.* (2007 ▶). For the structures of metal-organic compounds constructed by flexible bridging ligands, see: Zhang (2009*a* ▶,*b* ▶).
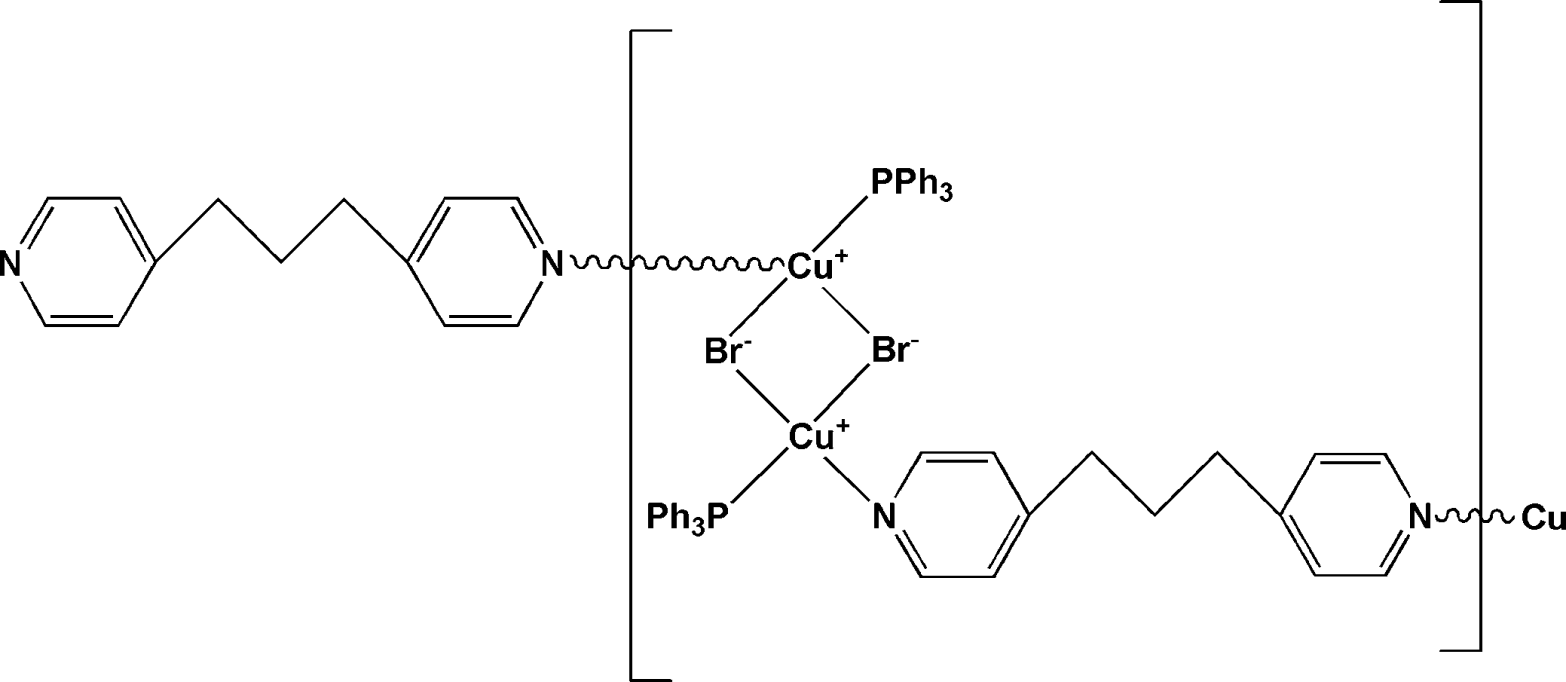


## Experimental

### 

#### Crystal data

[Cu_2_Br_2_(C_13_H_14_N_2_)(C_18_H_15_P)_2_]
*M* *_r_* = 1009.70
Monoclinic, 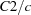

*a* = 25.703 (5) Å
*b* = 9.3679 (19) Å
*c* = 20.005 (4) Å
β = 111.58 (3)°
*V* = 4479.2 (18) Å^3^
*Z* = 4
Mo *K*α radiation
μ = 2.84 mm^−1^
*T* = 293 K
0.2 × 0.18 × 0.12 mm

#### Data collection

Rigaku Saturn 724+ (2 × 2 bin mode) diffractometer
Absorption correction: multi-scan (*CrystalClear*; Rigaku, 2008 ▶) *T*_min_ = 0.572, *T*_max_ = 0.711
10689 measured reflections
4450 independent reflections
3522 reflections with *I* > 2σ(*I*)
*R*_int_ = 0.031

#### Refinement

*R*[*F* ^2^ > 2σ(*F* ^2^)] = 0.047
*wR*(*F* ^2^) = 0.100
*S* = 1.08
4450 reflections
258 parameters
H-atom parameters constrained
Δρ_max_ = 0.50 e Å^−3^
Δρ_min_ = −0.40 e Å^−3^

### 

Data collection: *CrystalClear* (Rigaku, 2008 ▶); cell refinement: *CrystalClear*; data reduction: *CrystalClear*; program(s) used to solve structure: *SHELXTL* (Sheldrick, 2008 ▶); program(s) used to refine structure: *SHELXTL*; molecular graphics: *SHELXTL*; software used to prepare material for publication: *SHELXTL*.

## Supplementary Material

Crystal structure: contains datablock(s) I, global. DOI: 10.1107/S1600536812004084/rz2699sup1.cif

Structure factors: contains datablock(s) I. DOI: 10.1107/S1600536812004084/rz2699Isup2.hkl

Additional supplementary materials:  crystallographic information; 3D view; checkCIF report

## Figures and Tables

**Fig. 1.**
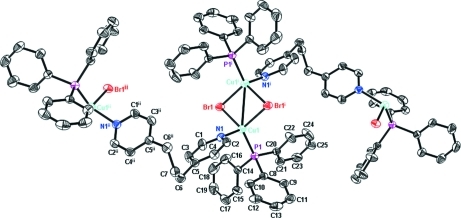
The molecular structure of title compound, with 30% probability displacement ellipsoids. H atoms have been omitted. Symmetry codes: (i) -x, -y, -z; (ii) -x, y, -z+1/2.

## supplementary crystallographic information

### Comment

The design and syntheses of metal-organic compounds have attracted great attention in recent years because of not only their intriguing architectures and topologies (Eddaoudi *et al.*, 2001) but also their potential applications (Banerjee *et al.*, 2008; Zhang *et al.*, 2007). Flexible bridging ligands can construct metal-organic compounds with various structures (Zhang, 2009*a*; Zhang, 2009*b*). The title compound {[CuBrP(Ph)_3_]_2_(bpp)}_n_ was constructed by the flexible bridging ligand 1,3-bis(pyridin-4-yl)propane (bpp) through diffusion reaction.

As illustrated in Fig. 1, each copper(I) atom is coordinated by two Br atoms, one P atom from a P(Ph)_3_ ligand and one N atom from a bpp molecule forming a distorted tetrahedral geometry. The dihedral angle formed by the pyridine rings of the bpp molecule is 34.59 (14)°. In the structure, two *µ*_2_-Br bridges connect two [CuP(Ph)_3_]^+^ units to form a neutral dimer [CuBrP(Ph)_3_]_2_; these dimers are then linked each other by the flexible bridging ligands bpp into one-dimensional chains parallel to the *c* axis.

### Experimental

A mixture of CuBr (1 mmol), P(Ph)_3_ (2 mmol) and *N*,*N*-dimethylformamide (dmf; 6 ml) was stirred for 5 minutes. After filtration, the colourless filtrate was carefully laid on the surface with dmf (1 ml) and a solution of bpp 0.5 (mmol) in *i*-PrOH (10 ml), in turn. Colourless block crystals were obtained after about five days.

### Refinement

H atoms were positioned geometrically and refined using a riding model, with C—H = 0.93–0.97 Å and with *U*_iso_(H) = 1.2 *U*_eq_(C).

### Crystal data

**Table:**

[Cu_2_Br_2_(C_13_H_14_N_2_)(C_18_H_15_P)_2_]	*F*(000) = 2040
*M**_r_* = 1009.70	*D*_x_ = 1.497 Mg m^−^^3^
Monoclinic, *C*2/*c*	Mo *K*α radiation, λ = 0.71073 Å
Hall symbol: -C 2yc	Cell parameters from 9037 reflections
*a* = 25.703 (5) Å	θ = 2.7–29.1°
*b* = 9.3679 (19) Å	µ = 2.84 mm^−^^1^
*c* = 20.005 (4) Å	*T* = 293 K
β = 111.58 (3)°	Block, colourless
*V* = 4479.2 (18) Å^3^	0.2 × 0.18 × 0.12 mm
*Z* = 4	

### Data collection

**Table:**

Rigaku Saturn 724+ (2x2 bin mode) diffractometer	4450 independent reflections
Radiation source: fine-focus sealed tube	3522 reflections with *I* > 2σ(*I*)
Graphite monochromator	*R*_int_ = 0.031
dtprofit.ref scans	θ_max_ = 26.4°, θ_min_ = 2.7°
Absorption correction: multi-scan (*CrystalClear*; Rigaku, 2008)	*h* = −21→31
*T*_min_ = 0.572, *T*_max_ = 0.711	*k* = −11→10
10689 measured reflections	*l* = −24→20

### Refinement

**Table:**

Refinement on *F*^2^	Primary atom site location: structure-invariant direct methods
Least-squares matrix: full	Secondary atom site location: difference Fourier map
*R*[*F*^2^ > 2σ(*F*^2^)] = 0.047	Hydrogen site location: inferred from neighbouring sites
*wR*(*F*^2^) = 0.100	H-atom parameters constrained
*S* = 1.08	*w* = 1/[σ^2^(*F*_o_^2^) + (0.040*P*)^2^ + 1.5813*P*] where *P* = (*F*_o_^2^ + 2*F*_c_^2^)/3
4450 reflections	(Δ/σ)_max_ < 0.001
258 parameters	Δρ_max_ = 0.50 e Å^−^^3^
0 restraints	Δρ_min_ = −0.40 e Å^−^^3^

### Special details

**Table:**

Geometry. All e.s.d.'s (except the e.s.d. in the dihedral angle between two l.s. planes) are estimated using the full covariance matrix. The cell e.s.d.'s are taken into account individually in the estimation of e.s.d.'s in distances, angles and torsion angles; correlations between e.s.d.'s in cell parameters are only used when they are defined by crystal symmetry. An approximate (isotropic) treatment of cell e.s.d.'s is used for estimating e.s.d.'s involving l.s. planes.
Refinement. Refinement of *F*^2^ against ALL reflections. The weighted *R*-factor *wR* and goodness of fit *S* are based on *F*^2^, conventional *R*-factors *R* are based on *F*, with *F* set to zero for negative *F*^2^. The threshold expression of *F*^2^ > σ(*F*^2^) is used only for calculating *R*-factors(gt) *etc*. and is not relevant to the choice of reflections for refinement. *R*-factors based on *F*^2^ are statistically about twice as large as those based on *F*, and *R*- factors based on ALL data will be even larger.

### Fractional atomic coordinates and isotropic or equivalent isotropic displacement parameters (Å^2^)

**Table:**

	*x*	*y*	*z*	*U*_iso_*/*U*_eq_	Occ. (<1)
Br1	0.037465 (15)	−0.13108 (4)	0.085054 (19)	0.05206 (14)	
Cu1	0.045283 (18)	0.10762 (4)	0.03058 (2)	0.05009 (16)	
P1	0.12847 (4)	0.14974 (9)	0.02434 (5)	0.0420 (2)	
N1	0.02809 (12)	0.2487 (3)	0.09919 (15)	0.0480 (7)	
C1	0.05533 (18)	0.2313 (4)	0.16946 (19)	0.0646 (11)	
H1A	0.0706	0.1420	0.1857	0.078*	
C2	0.00515 (15)	0.3770 (4)	0.0794 (2)	0.0516 (9)	
H2A	−0.0154	0.3921	0.0308	0.062*	
C3	0.06232 (18)	0.3374 (4)	0.2197 (2)	0.0646 (11)	
H3A	0.0820	0.3186	0.2681	0.078*	
C4	0.00981 (15)	0.4879 (4)	0.12585 (18)	0.0540 (9)	
H4A	−0.0075	0.5746	0.1085	0.065*	
C5	0.04018 (15)	0.4712 (4)	0.19846 (18)	0.0481 (8)	
C6	0.05047 (16)	0.5895 (4)	0.2525 (2)	0.0595 (10)	
H6A	0.0778	0.6541	0.2462	0.071*	
H6B	0.0672	0.5482	0.3002	0.071*	
C7	0.0000	0.6766 (5)	0.2500	0.0598 (14)	
H7A	0.0109	0.7378	0.2920	0.072*	0.50
H7B	−0.0109	0.7378	0.2080	0.072*	0.50
C8	0.13901 (13)	0.3398 (3)	0.01233 (18)	0.0431 (8)	
C9	0.15024 (16)	0.3953 (4)	−0.0452 (2)	0.0564 (10)	
H9A	0.1547	0.3340	−0.0793	0.068*	
C10	0.13194 (15)	0.4350 (4)	0.0618 (2)	0.0560 (9)	
H10A	0.1242	0.4000	0.1007	0.067*	
C11	0.15479 (18)	0.5417 (4)	−0.0523 (2)	0.0718 (12)	
H11A	0.1630	0.5777	−0.0906	0.086*	
C12	0.13618 (17)	0.5808 (4)	0.0539 (3)	0.0719 (12)	
H12A	0.1315	0.6431	0.0875	0.086*	
C13	0.14731 (18)	0.6335 (4)	−0.0035 (3)	0.0767 (14)	
H13A	0.1498	0.7316	−0.0091	0.092*	
C14	0.19053 (15)	0.1027 (4)	0.10280 (18)	0.0468 (8)	
C15	0.23762 (16)	0.1870 (4)	0.1286 (2)	0.0644 (10)	
H15A	0.2380	0.2747	0.1070	0.077*	
C16	0.19065 (18)	−0.0256 (5)	0.1358 (2)	0.0791 (13)	
H16A	0.1590	−0.0833	0.1196	0.095*	
C17	0.28428 (18)	0.1441 (5)	0.1858 (2)	0.0786 (13)	
H17A	0.3158	0.2021	0.2023	0.094*	
C18	0.2374 (2)	−0.0702 (6)	0.1931 (3)	0.1048 (18)	
H18A	0.2373	−0.1582	0.2145	0.126*	
C19	0.2840 (2)	0.0159 (7)	0.2183 (2)	0.0931 (16)	
H19A	0.3152	−0.0131	0.2572	0.112*	
C20	0.14205 (14)	0.0655 (3)	−0.04961 (17)	0.0440 (8)	
C21	0.19394 (16)	0.0172 (4)	−0.0453 (2)	0.0603 (10)	
H21A	0.2242	0.0236	−0.0020	0.072*	
C22	0.09837 (18)	0.0530 (5)	−0.1144 (2)	0.0756 (13)	
H22A	0.0627	0.0809	−0.1180	0.091*	
C23	0.20140 (18)	−0.0412 (5)	−0.1054 (2)	0.0744 (12)	
H23A	0.2364	−0.0753	−0.1016	0.089*	
C24	0.1062 (2)	0.0002 (6)	−0.1743 (2)	0.1008 (18)	
H24A	0.0764	−0.0025	−0.2182	0.121*	
C25	0.1577 (2)	−0.0482 (5)	−0.1693 (2)	0.0817 (14)	
H25A	0.1628	−0.0858	−0.2095	0.098*	

### Atomic displacement parameters (Å^2^)

**Table:**

	*U*^11^	*U*^22^	*U*^33^	*U*^12^	*U*^13^	*U*^23^
Br1	0.0493 (2)	0.0439 (2)	0.0592 (3)	−0.00242 (15)	0.01535 (18)	0.00777 (16)
Cu1	0.0479 (3)	0.0470 (3)	0.0577 (3)	−0.00363 (19)	0.0221 (2)	−0.0010 (2)
P1	0.0399 (5)	0.0390 (5)	0.0458 (5)	−0.0023 (4)	0.0142 (4)	−0.0011 (4)
N1	0.0500 (18)	0.0442 (17)	0.0510 (17)	0.0021 (13)	0.0199 (14)	0.0027 (14)
C1	0.093 (3)	0.046 (2)	0.052 (2)	0.019 (2)	0.023 (2)	0.011 (2)
C2	0.051 (2)	0.053 (2)	0.047 (2)	0.0040 (17)	0.0133 (17)	0.0068 (18)
C3	0.083 (3)	0.060 (2)	0.045 (2)	0.016 (2)	0.017 (2)	0.004 (2)
C4	0.059 (2)	0.041 (2)	0.059 (2)	0.0082 (17)	0.0188 (18)	0.0035 (19)
C5	0.046 (2)	0.049 (2)	0.052 (2)	−0.0021 (16)	0.0211 (16)	−0.0025 (18)
C6	0.060 (3)	0.055 (2)	0.064 (2)	−0.0078 (19)	0.023 (2)	−0.0077 (19)
C7	0.081 (4)	0.044 (3)	0.056 (3)	0.000	0.026 (3)	0.000
C8	0.0358 (19)	0.0414 (18)	0.0485 (19)	−0.0005 (14)	0.0115 (15)	0.0021 (16)
C9	0.056 (2)	0.051 (2)	0.058 (2)	−0.0036 (17)	0.0167 (19)	0.0050 (18)
C10	0.056 (2)	0.048 (2)	0.068 (2)	−0.0072 (17)	0.0279 (19)	−0.0071 (19)
C11	0.073 (3)	0.056 (3)	0.077 (3)	−0.007 (2)	0.016 (2)	0.020 (2)
C12	0.061 (3)	0.045 (2)	0.108 (4)	−0.0040 (19)	0.029 (3)	−0.018 (2)
C13	0.068 (3)	0.037 (2)	0.113 (4)	−0.0019 (19)	0.018 (3)	0.011 (3)
C14	0.045 (2)	0.048 (2)	0.050 (2)	0.0023 (16)	0.0201 (17)	0.0030 (17)
C15	0.056 (3)	0.061 (2)	0.065 (2)	−0.003 (2)	0.010 (2)	−0.003 (2)
C16	0.060 (3)	0.077 (3)	0.092 (3)	0.003 (2)	0.018 (2)	0.030 (3)
C17	0.051 (3)	0.104 (4)	0.064 (3)	0.003 (2)	0.002 (2)	−0.011 (3)
C18	0.082 (4)	0.119 (4)	0.104 (4)	0.017 (3)	0.023 (3)	0.066 (4)
C19	0.066 (3)	0.139 (5)	0.063 (3)	0.029 (3)	0.010 (2)	0.024 (3)
C20	0.045 (2)	0.0386 (18)	0.0489 (19)	−0.0027 (15)	0.0177 (16)	−0.0034 (16)
C21	0.049 (2)	0.068 (3)	0.066 (2)	−0.0080 (19)	0.0240 (19)	−0.014 (2)
C22	0.056 (3)	0.096 (3)	0.065 (2)	0.016 (2)	0.010 (2)	−0.032 (2)
C23	0.056 (3)	0.085 (3)	0.092 (3)	−0.006 (2)	0.040 (3)	−0.022 (3)
C24	0.073 (4)	0.142 (5)	0.073 (3)	0.012 (3)	0.011 (3)	−0.052 (3)
C25	0.076 (3)	0.101 (4)	0.073 (3)	−0.008 (3)	0.033 (3)	−0.038 (3)

### Geometric parameters (Å, º)

**Table:**

Br1—Cu1^i^	2.5108 (12)	C10—C12	1.384 (5)
Br1—Cu1	2.5276 (7)	C10—H10A	0.9300
Cu1—N1	2.067 (3)	C11—C13	1.368 (6)
Cu1—P1	2.2226 (11)	C11—H11A	0.9300
Cu1—Br1^i^	2.5108 (12)	C12—C13	1.373 (6)
Cu1—Cu1^i^	2.9810 (10)	C12—H12A	0.9300
P1—C20	1.820 (3)	C13—H13A	0.9300
P1—C8	1.830 (3)	C14—C16	1.371 (5)
P1—C14	1.833 (4)	C14—C15	1.377 (5)
N1—C1	1.330 (4)	C15—C17	1.378 (5)
N1—C2	1.333 (4)	C15—H15A	0.9300
C1—C3	1.378 (5)	C16—C18	1.385 (6)
C1—H1A	0.9300	C16—H16A	0.9300
C2—C4	1.370 (5)	C17—C19	1.365 (7)
C2—H2A	0.9300	C17—H17A	0.9300
C3—C5	1.377 (5)	C18—C19	1.377 (7)
C3—H3A	0.9300	C18—H18A	0.9300
C4—C5	1.382 (5)	C19—H19A	0.9300
C4—H4A	0.9300	C20—C22	1.373 (5)
C5—C6	1.502 (5)	C20—C21	1.381 (5)
C6—C7	1.518 (5)	C21—C23	1.396 (5)
C6—H6A	0.9700	C21—H21A	0.9300
C6—H6B	0.9700	C22—C24	1.378 (5)
C7—C6^ii^	1.518 (5)	C22—H22A	0.9300
C7—H7A	0.9700	C23—C25	1.358 (6)
C7—H7B	0.9700	C23—H23A	0.9300
C8—C9	1.387 (5)	C24—C25	1.369 (6)
C8—C10	1.393 (5)	C24—H24A	0.9300
C9—C11	1.389 (5)	C25—H25A	0.9300
C9—H9A	0.9300		
			
Cu1^i^—Br1—Cu1	72.547 (19)	C8—C9—H9A	119.8
N1—Cu1—P1	111.65 (8)	C11—C9—H9A	119.8
N1—Cu1—Br1^i^	103.91 (8)	C12—C10—C8	121.1 (4)
P1—Cu1—Br1^i^	116.05 (4)	C12—C10—H10A	119.5
N1—Cu1—Br1	102.07 (8)	C8—C10—H10A	119.5
P1—Cu1—Br1	114.33 (3)	C13—C11—C9	120.6 (4)
Br1^i^—Cu1—Br1	107.453 (19)	C13—C11—H11A	119.7
N1—Cu1—Cu1^i^	112.31 (8)	C9—C11—H11A	119.7
P1—Cu1—Cu1^i^	135.99 (4)	C13—C12—C10	119.9 (4)
Br1^i^—Cu1—Cu1^i^	53.99 (2)	C13—C12—H12A	120.0
Br1—Cu1—Cu1^i^	53.47 (2)	C10—C12—H12A	120.0
C20—P1—C8	103.50 (16)	C11—C13—C12	119.9 (4)
C20—P1—C14	103.00 (16)	C11—C13—H13A	120.0
C8—P1—C14	102.83 (15)	C12—C13—H13A	120.0
C20—P1—Cu1	116.40 (12)	C16—C14—C15	118.4 (3)
C8—P1—Cu1	111.84 (11)	C16—C14—P1	118.1 (3)
C14—P1—Cu1	117.48 (12)	C15—C14—P1	123.5 (3)
C1—N1—C2	115.3 (3)	C14—C15—C17	121.4 (4)
C1—N1—Cu1	117.7 (2)	C14—C15—H15A	119.3
C2—N1—Cu1	123.9 (2)	C17—C15—H15A	119.3
N1—C1—C3	124.0 (3)	C14—C16—C18	120.7 (4)
N1—C1—H1A	118.0	C14—C16—H16A	119.7
C3—C1—H1A	118.0	C18—C16—H16A	119.7
N1—C2—C4	124.3 (3)	C19—C17—C15	119.7 (4)
N1—C2—H2A	117.8	C19—C17—H17A	120.1
C4—C2—H2A	117.8	C15—C17—H17A	120.1
C5—C3—C1	120.2 (3)	C19—C18—C16	120.0 (5)
C5—C3—H3A	119.9	C19—C18—H18A	120.0
C1—C3—H3A	119.9	C16—C18—H18A	120.0
C2—C4—C5	120.1 (3)	C17—C19—C18	119.8 (4)
C2—C4—H4A	119.9	C17—C19—H19A	120.1
C5—C4—H4A	119.9	C18—C19—H19A	120.1
C3—C5—C4	115.9 (3)	C22—C20—C21	117.8 (3)
C3—C5—C6	120.4 (3)	C22—C20—P1	118.0 (3)
C4—C5—C6	123.7 (3)	C21—C20—P1	124.1 (3)
C5—C6—C7	116.8 (3)	C20—C21—C23	120.7 (4)
C5—C6—H6A	108.1	C20—C21—H21A	119.7
C7—C6—H6A	108.1	C23—C21—H21A	119.7
C5—C6—H6B	108.1	C20—C22—C24	121.4 (4)
C7—C6—H6B	108.1	C20—C22—H22A	119.3
H6A—C6—H6B	107.3	C24—C22—H22A	119.3
C6^ii^—C7—C6	114.9 (4)	C25—C23—C21	120.2 (4)
C6^ii^—C7—H7A	108.5	C25—C23—H23A	119.9
C6—C7—H7A	108.5	C21—C23—H23A	119.9
C6^ii^—C7—H7B	108.5	C25—C24—C22	120.2 (4)
C6—C7—H7B	108.5	C25—C24—H24A	119.9
H7A—C7—H7B	107.5	C22—C24—H24A	119.9
C9—C8—C10	118.1 (3)	C23—C25—C24	119.6 (4)
C9—C8—P1	124.0 (3)	C23—C25—H25A	120.2
C10—C8—P1	117.8 (3)	C24—C25—H25A	120.2
C8—C9—C11	120.4 (4)		

Symmetry codes: (i) −*x*, −*y*, −*z*; (ii) −*x*, *y*, −*z*+1/2.
